# Cerium Oxide Nanoparticles Promote Osteoplastic Precursor Differentiation by Activating the Wnt Pathway

**DOI:** 10.1007/s12011-022-03168-9

**Published:** 2022-03-01

**Authors:** Junchao Luo, Senbo Zhu, Yu Tong, Yin Zhang, Yong Li, Li Cao, Mingxiang Kong, Min Luo, Qing Bi, Qiong Zhang

**Affiliations:** 1 Department of Orthopedics, Zhejiang Provincial People’s Hospital, Hangzhou, 310014 Zhejiang China; 2 Department of Orthopedics, The Second Affiliated Hospital of Wenzhou Medical University, Wenzhou, Zhejiang China 325027; 3 Zhejiang University of Technology, Hangzhou, 310014 Zhejiang China; 4 Bengbu Medical College, Bengbu, 233030 Anhui China; 5 Dongyang Hospital of Traditional Chinese Medicine, Jinghua, 322199 Zhejiang China; 6 Institute of Sports Medicine and Osteoarthropathy of Hangzhou Medical College, 481# Binwen Road, Hangzhou, 310000 Zhejiang China

**Keywords:** Nanoparticle, MC3T3-E1 cells, Osteogenesis differentiation, RNA-seq

## Abstract

Osteoplastic precursors are critical for fracture repair and bone homeostasis maintenance. Cerium oxide nanoparticles (CeO_2_ NPs) can promote the osteogenic differentiation of mesenchymal stem cells and secrete vascular endothelial growth factors. However, little is known about its role in precursor osteoblasts; therefore, we further investigated the effect and mechanism of CeO_2_ NPs in precursor osteoblasts. Cell counting kit-8 analysis was utilized to detect the toxicity of CeO_2_ NPs on MC3T3-E1 mouse osteogenic precursor cells. Then, alizarin red S staining was employed to assess the degree of extracellular matrix mineralization, and quantitative real-time polymerase chain reaction analysis was performed to measure the levels of osteogenesis-related genes. To identify differentially expressed genes, mRNA-sequencing was performed. Subsequently, GO and KEGG analyses were deployed to identify the major downstream pathways, whereas Western blot was used for verification. CeO_2_ NPs significantly enhanced the ability of MC3T3-E1 precursor osteoblasts to enhance matrix mineralization and increased the expression of osteogenic genes such as runt-related transcription factor 2, collagen Iα1, and osteocalcin. Pathway analysis revealed that CeO_2_ NPs enhanced the nuclear translocation of β-catenin and activated the Wnt pathway by promoting family with sequence similarity 53 member B/simplet expression, while Western blot analysis indicated the same results. After using a Wnt pathway inhibitor (KYA1797K), the simulative effect of CeO_2_ NPs was abolished. This study revealed that CeO_2_ NPs promoted MC3T3-E1 precursor osteoblast differentiation by activating the Wnt pathway.

## Introduction

Osteoblast precursors play a significant role in fracture repair and bone homeostasis maintenance. Maes et al. disclosed that osteoblast precursors and vascular endothelial cells showed co-invasion during fracture healing in mice, indicating that osteoblast precursors are involved in fracture healing [1]. Age-related bone loss is intimately linked to imbalances in bone formation and resorption [2]. Mesenchymal stem cells, distributed in bone marrow and other body parts, follow the blood circulation to reach the bone surface and differentiate into precursors osteoblasts, which further form mature osteoblasts, produce bone matrix, and promote matrix mineralization [3]. Therefore, reduced differentiation of precursor osteoblasts decreased bone formation, leading to osteoporosis. Promoting differentiation of precursor osteoblasts is an important goal for fracture repair and osteoporosis treatment.

Nanoparticles are considered promising in diagnosing and treating diseases. Passive nanoparticles (NPs) have recently been approved by the US Food and Drug Administration (FDA) for the clinical treatment of cancer [4, 5]. Kalyanaraman et al. evaluated the safety of cerium oxide nanoparticles (CeO_2_ NPs) by implanting 250–1000 mg CeO_2_ NPs subcutaneously in rats and observing changes in local tissues and major organs after 28 days [6]. The results demonstrated that CeO_2_ NPs did not significantly stimulate local tissues and showed no toxicity to major organs, implying that cerium oxide nanoparticles have good biosafety. Previous studies have demonstrated that CeO_2_ NPs are characterized by excellent scavenging ability of active free radicals, mainly based on their potential to simulate glutathione peroxidase and catalase during the transformation of Ce^3+^ and Ce^4+^ valence states [7]. Li et al. found that CeO_2_ NPs reversed the decrease of superoxide dismutase activity in H_2_O_2_-induced osteoblast oxidative damage and reduced the production of reactive oxygen species and malondialdehyde formation [8]. Wei et al. suggested that CeO_2_ NPs can induce M2-type differentiation of macrophages and promote osteogenic differentiation of mesenchymal stem cells [9]. Similarly, Purohit et al. stated that composite scaffolds with CeO_2_ NPs significantly promoted bone repair [10]. However, the mechanism by which CeO_2_ NPs promote osteoblast differentiation has not been fully elucidated.

In this study, we determined the effects of CeO_2_ NPs on MC3T3-E1 mouse precursor osteoblasts. It was revealed that CeO_2_ NPs promote the nuclear translocation of β-catenin protein, activate the Wnt pathway, and promote the differentiation of precursor osteoblasts by increasing Fam53B expression.

## Material and Methods

### Preparation and Characterization of Cerium Oxide Nanoparticles

Cerium nitrate hexahydrate was added to a mixture of ethylene glycol and double-distilled water and heated to 60 ℃. After adding ammonia, the solution was then agitated until it turned yellowish-white. CeO_2_ NPs were obtained by high-speed centrifugation. After washing three times, sodium citrate was employed as a surfactant to activate NPs. Dynamic light scattering (DLS) analysis was utilized to evaluate particle size and zeta potential. Transmission electron microscopy (TEM) was used to observe the morphology of CeO_2_ NPs. X-ray powder diffraction patterns were used to evaluate the crystallinity of NPs. X-ray photoelectron spectroscopy was utilized to assess the chemical composition of the surface and valence state of CeO_2_ NPs.

### Cell Culture

MC3T3-E1 mouse precursor osteoblasts cells (American Type Culture Collection) were cultured with Dulbecco’s Modified Eagle Medium (HyClone) supplemented with 10% fetal bovine serum, 100 IU/mL penicillin, and 100 µg/mL streptomycin (Invitrogen) at 37 ℃ with 5% CO_2_. The medium was replaced after 2–3 days.

### Cytotoxicity Test

Cytotoxicity tests were utilized to find suitable concentrations of CeO_2_ NPs. MC3T3-E1 precursor osteoblasts were inoculated into 96-well plates at a density of 5 × 10^3^ cells/well, and CeO_2_ NPs at different concentrations (0, 1, 3, 10, 30, 50, 100, 150, and 200 µg/mL) at 37 ℃ for 24 and 48 h, respectively. After discarding the medium, it is washed once with PBS. The absorbance value at 450 nm was detected after incubating with non-haematine medium containing 10% cell counting kit-8 reagent (Dojindo) at 37 ℃ for 2 h and 30 min.

### Alizarin Red S Staining

The impact of CeO_2_ NPs on the mineralization of MC3T3-E1 precursor osteoblasts was evaluated using alizarin red S staining. After covering the 12-well plates with gelatin, precursor osteoblasts were seeded into the plates at a density of 3 × 10^4^ cells/mL. Precursor osteoblasts were cultured in osteogenic differentiation induction media containing CeO_2_ NPs at different concentrations (0, 1, and 3 µg/mL). After 14 days of differentiation, precursor osteoblasts were fixed with 4% paraformaldehyde phosphate buffer for 15 min. We washed the cells with ddH_2_O and stained them with alizarin red S (Sigma) for 3 min. The image was captured after the sample was rinsed twice. The procedures were independently repeated three times to ensure reliability.

### Quantitative Real-Time Polymerase Chain Reaction Analysis

The effect of CeO_2_ NPs on osteogenesis differentiation of precursor osteoblasts was further analyzed by quantitative real-time polymerase chain reaction analysis (qRT-PCR) according to the published protocol [11]. We used RNA-quick purification kit (ESscience) to extract total cellular RNA. The cDNA was synthesized using 1 µg of RNA utilizing a PrimescriptTM RT reagent kit with a gDNA eraser (Takara Bioindustry) according to manufacturer’s protocol. cDNA was diluted with nuclease-free water in a ratio of 1:10. Maxima SYBR green PCR master mix (2 ×) was used for real-time PCR analysis. The program used for real-time PCR included initial denaturation at 95 ℃ for 2 min, followed by 40 cycles of denaturation at 95 ℃ for 15 s, annealing at 60 ℃ for 30 s and extension at 72 ℃ for 30 s. Target gene expression was normalized to GAPDH expression, and 2^−ΔΔCT^ method was utilized to determine relative gene expression. The primers of runt related transcription factor 2 (Runx2), osteocalcin (OCN), collagen Iα1 (Col Iα1), family with sequence similarity 53 member B (Fam53B), and GAPDH were designed with the aid of NCBI Primer-Blast Tool, which were listed as follows: Runx2 (F) 5′-AGAGTCAGATTACAGATCCCAGG-3′, (R) 5′-TGGCTCTTCTTACTGAGAGAGG-3′; OCN (F) 5′-TAGTGAACAGACTCCGGCGCTA-3′, (R) 5′-TGTAGGCGGTCTTCAAGCCAT-3′; Col Iα1 (F) 5′-GCTTCACCTACAGCACCCTTGT-3′, (R) 5′-TGACTGTCTTGCCCCAAGTTC-3′; Fam53B (F) 5′-ACCTGGATAGAAAATGCCCTCT-3′, (R)5′-GAGGTCTTTGATTAGGCTGGTC-3′; GAPDH (F) 5′-TGACCTCAACTACATGGTCTACA-3′, (R) 5′-CTTCCCATTCTCGGCCTTG-3′.

### mRNA-Sequencing

Total cellular RNA was extracted following the same aforementioned method. Messenger (m)RNA for each sample was enriched using poly-T oligo-attached magnetic beads. The first strand cDNA was synthesized using random hexamer primer and RNase H. Second strand cDNA synthesis was subsequently performed using buffer, dNTPs, DNA polymerase I, and RNase H. The purified cDNA was subjected to a final reparation, the addition of an A-tailing, and an adapter. Ultimately, cDNA library was sequenced on Illumina Hiseq xTen platform.

### Western Blot Analysis

Western blotting analysis was performed as described previously [12]. Total protein was extracted by direct lysis of cells using cold RIPA lysis buffer containing 1 mmol/L phenylmethylsulfonyl fluoride. Nuclear protein was extracted using a nuclear protein extraction kit (Solarbio) following manufacturer’s protocol. The protein concentration was determined using a Pierce™ BCA protein assay kit (Thermo Scientific). Equal amounts of protein (20 µg) were fractionated on precast protein improve gels (Yeasen Biotechnology) and transferred to a PVDF membrane (Merck Millipore). The membrane was then blocked with 5% nonfat dry milk at room temperature for 2 h. After three additional washes with TBST, membranes were incubated overnight with primary antibodies at 4 ℃. The primary antibodies are as follows: Runx2 (mouse mAb; cat. no. ab76956; Abcam), OCN (rabbit mAb; cat. no. ab93876; Abcam), Fam53B (rabbit mAb; cat. no. NBP1-88,976; Novus), GAPDH (mouse mAb; cat. no. ab8245; Abcam), β-catenin (rabbit mAb; cat. no. 51067–2-AP; Proteintech), lamin A/C (rabbit mAb; cat. no. 51067–2-AP; Proteintech). After washing three times with TBST for 10 min, membranes were then incubated with HRP-conjugated secondary antibodies (goat Anti-Rabbit IgG, Cat.no.ZB-2301, Zhongshan Golden Bridge Biotechnology; goat Anti-Mouse IgG, cat. no. A0216, Beyotime Institute of Biotechnology) for 2 h at room temperature. After being washed three more times with TBST, protein bands were visualized using an ECL kit (FDbio Science Biotechnology).

### Statistical Analysis

All data are presented as mean ± standard deviation (SD). The data comparisons between multiple groups were analyzed using one-way analysis of variance (ANOVA), whereas comparisons between two groups were analyzed using Student’s *t*-test. A *p*-value less than 0.05 was considered significant. During the differential expression analysis, all *p*-values were adjusted using Benjamini and Hochberg method. Genes with *q* ≤ 0.05 and |log_2_Ratio|≥ 1 are identified as differentially expressed genes (DEGs). GO enrichment of DEGs was implemented by the hypergeometric test, in which *p*-value is calculated and adjusted as a *q*-value. GO terms with *q* < 0.05 were considered significantly enriched. KEGG enrichment of DEGs was performed using hypergeometric test, in which *p*-value was adjusted by multiple comparisons as *q*-value, which was considered significantly enriched when *p*-value was less than 0.05. Differential expression analysis was performed using DEGseq R package (v1.16), and other analyses were performed using SPSS program version 23.0 (IBM Corp., Armonk, NY).

## Results

### Characterization of Cerium Oxide Nanoparticles

**Fig. 1:**
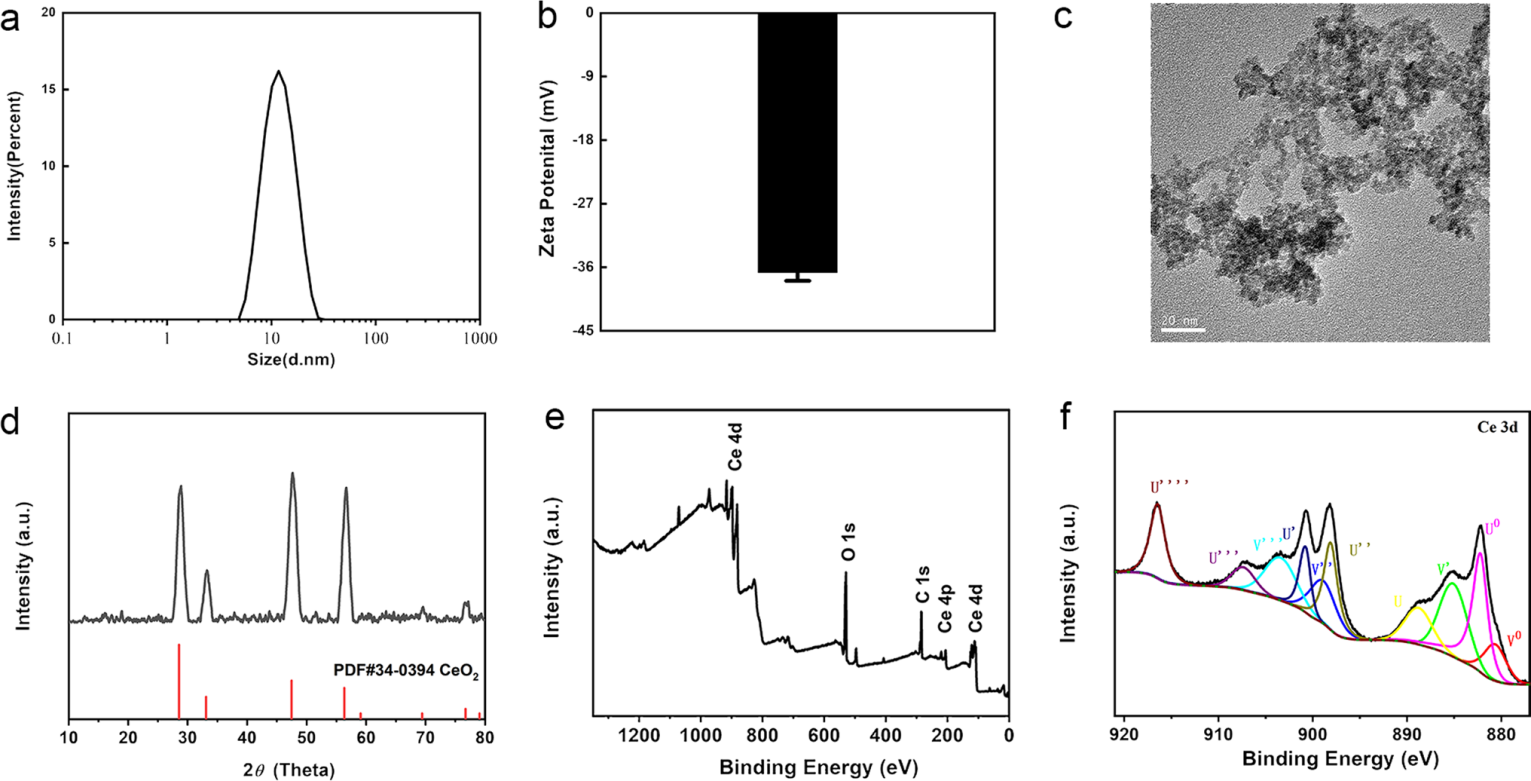
Characterization of cerium oxide nanoparticles. **A** Particle size distribution. **B** Zeta potential. **C** A transmission electron microscopy image. **D** X-ray powder diffraction patterns for CeO_2_ NPs. **E** X-ray photoelectron spectroscopy spectra of CeO_2_ NPs. **F** Development of Ce 3d X-ray photoelectron spectroscopy spectra and fitted Ce 3d X-ray photoelectron spectroscopy spectra

The results of DLS revealed that the mean particle size and zeta potential of CeO_2_ NPs were 12.13 nm and − 36 mV, respectively (Fig. 1a–b). TEM demonstrated that most nanoparticles were oval in shape (Fig. 1c). The diffraction peaks from powder X-ray diffraction patterns were well matched to the standard cerium oxide pattern (PDF2:34–0394), indicating the high purity of our synthetic nanoparticles (Fig. 1d). X-ray photoelectron spectroscopy revealed that the relative levels of Ce^4+^ and Ce^3+^ were 42.13% and 57.87% (Fig. 1e–f).

### Cerium Oxide Nanoparticles Promoted Precursor Osteoblast Differentiation in MC3T3-E1 Cells

**Fig. 2:**
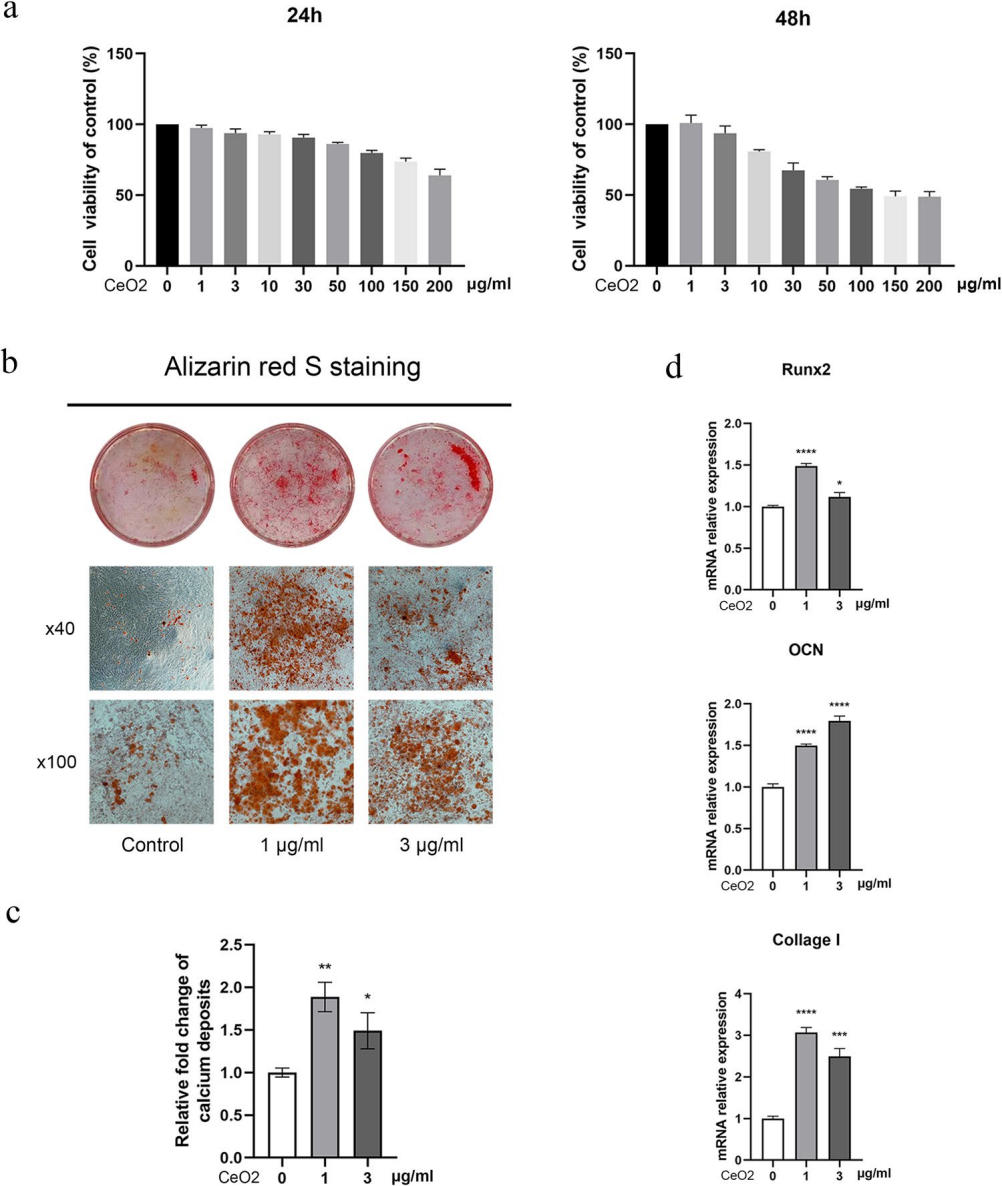
Cerium oxide nanoparticles increased osteogenic differentiation of MC3T3-E1 precursor osteoblast. **A** Effect of cerium oxide nanoparticles on cell viability of MC3T3-E1 cells. **B** Alizarin red S staining was performed on days 14 after treatment with cerium oxide nanoparticles. **C** Semi-quantitative analysis of calcium deposits. **D** qRT-PCR was employed to examine changes in the levels of Runx2, osteocalcin, and collagen Iα1 in MC3T3-E1 cells after treatment with cerium oxide nanoparticles for 48 h

CCK-8 analysis indicated that CeO_2_ NPs had a non-significant effect on chondrocyte viability in the range of 0–3 µg/mL (Fig. 2a). Consequently, we used concentrations of 1 and 3 µg/mL NPs for subsequent in vitro experiments. Then, Alizarin red staining and qPCR were utilized to determine the effect of CeO_2_ NPs on osteogenic differentiation of MC3T3-E1 cells. As displayed in Fig. 2b–c, we detected a significant increase in Ca^2+^ accumulation in the extracellular matrix. Among them, CeO_2_ NP-mineralized nodules at a 1 µg/ml concentration were the most significant. Additionally, we conducted qPCR to examine the expression of osteogenic marker genes (Runx2, Col Iα1 and OCN) in MC3T3-E1 cells and the effect of CeO_2_ NPs on enhancing osteogenesis. Compared with the control group, MC3T3-E1 cells treated with CeO_2_ NPs for 48 h showed significantly enhanced osteogenic differentiation (Fig. 2d).

### Functional Annotation and Pathways Analysis of Differential Expressed Genes

**Fig. 3:**
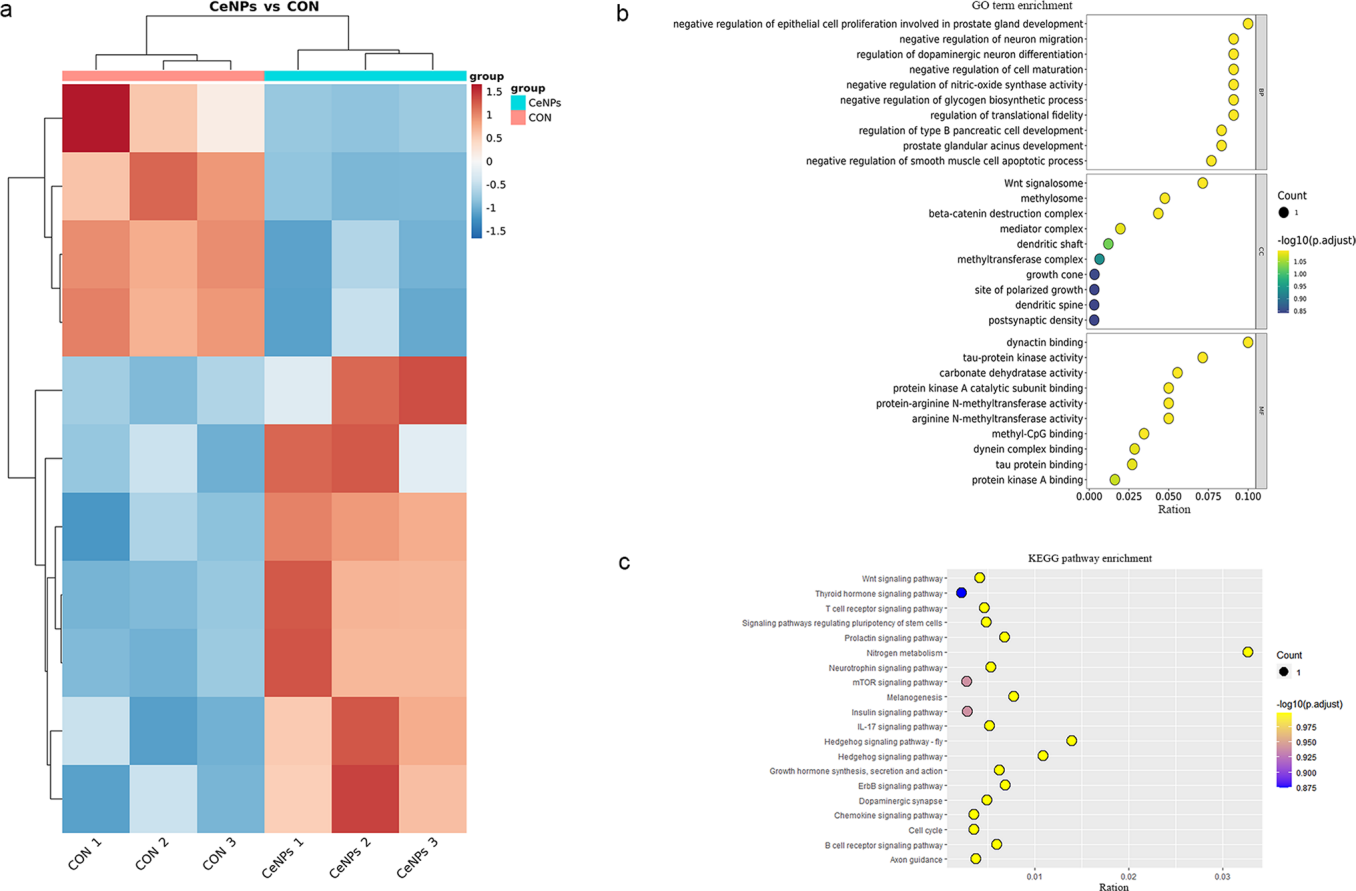
Heatmap and enrichment analysis of cerium oxide nanoparticles induced by differential gene expression. **A** Heatmap. **B** GO term enrichment. **c** KEGG pathway enrichment

Considering that 1 µg/mL CeO_2_ NPs demonstrated the best effect, we used this concentration in subsequent experiments. MC3T3-31 mouse precursor osteoblasts were treated with 1 µg/mL NPs for 48 h, and RNA of it and control group were extracted for sequencing. The results revealed that seven DEGs were upregulated, and four were downregulated compared with the control group. Heatmap indicated that DEGs had similar expression patterns within groups and different expression patterns between groups (Fig. 3a). These DEGs were significantly enriched in 30 GO items, such as negative regulation of nitric-oxide synthase activity in biological process and Wnt signalosome in cell composition (*q* < 0.05) (Fig. 3b). Furthermore, 20 KEGG pathways were substantially enriched, including nitrogen metabolism, prolactin signaling pathway, growth hormone synthesis, Wnt pathway, insulin signaling pathway, and thyroid hormone signaling pathway (Fig. 3c).

Wnt pathway activation is critical in differentiating precursor osteoblasts. As depicted in Fig. 3a, Fam53B gene expression was substantially upregulated in MC3T3-E1 cells treated with CeO_2_ NPs. The Simplet/Fam53B belongs to a family of proteins (Fam53A, Fam53B, and Fam53C), and previous studies have revealed its involvement in Wnt pathway transduction by promoting β-catenin nuclear translocation [13].

### Fam53B Activates the Wnt Pathway by Promoting Beta-Catenin Nuclear Translocation

**Fig. 4:**
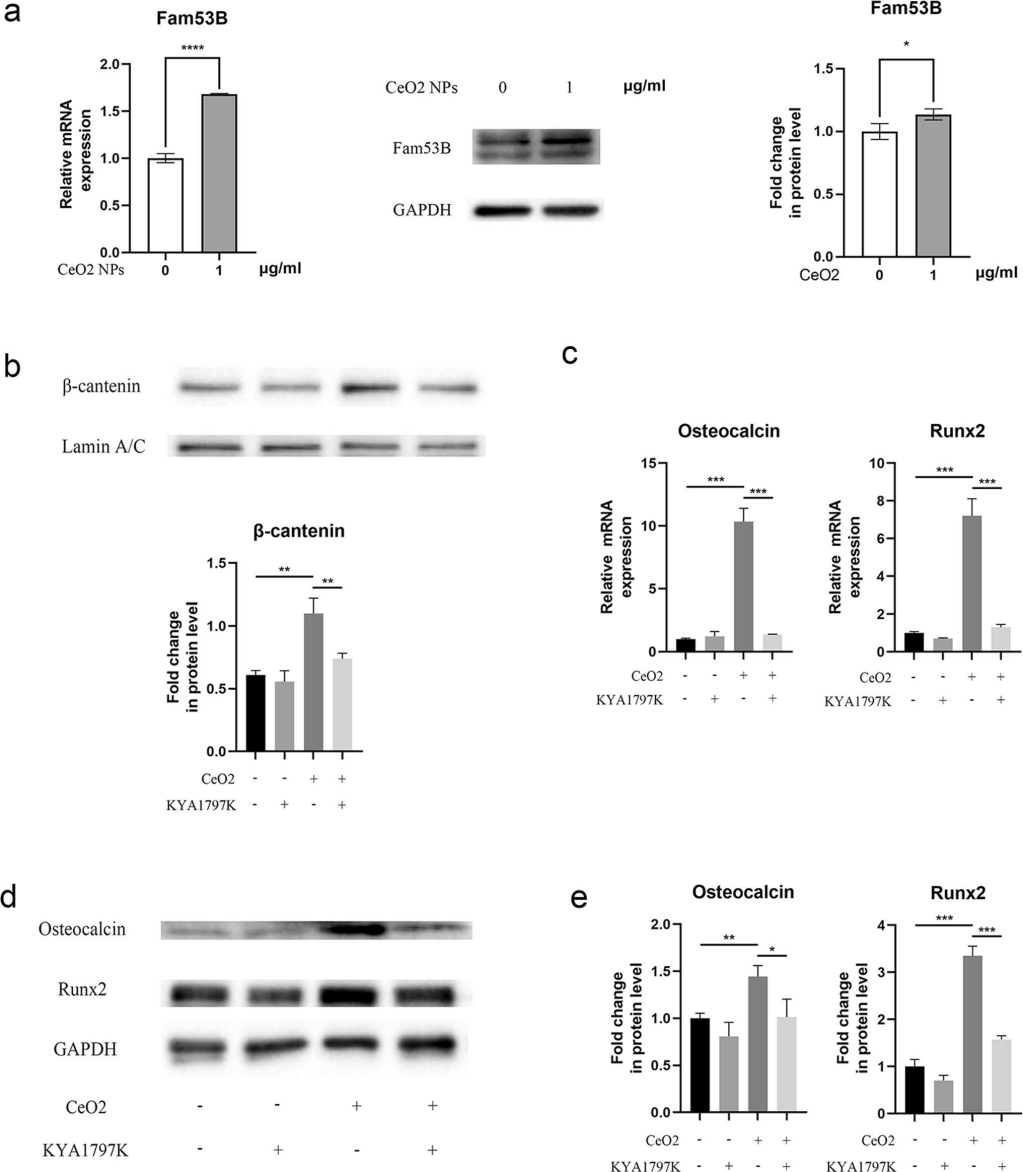
Cerium oxide nanoparticles activated Wnt/β-catenin signaling to promote osteogenesis differentiation by increasing Fam53B expression. **A** Fam53B expression in MC3T3-E1 treated with cerium oxide nanoparticles for 48 h using qRT-PCR, Western blotting, and quantification related to GAPDH levels. **B** The levels of β-catenin protein in the nucleus of MC3T3-E1 treated with 1 µg/mL cerium oxide nanoparticles in the presence or absence of KYA1797K after 48 h. **C** qRT-PCR was used to examine changes in the levels of Runx2, osteocalcin in MC3T3-E1 treated with cerium oxide nanoparticles in the presence or absence of KYA1797K after 48 h. **D** Representative Western blot images of Runx2 and osteocalcin in MC3T3-E1 treated as described above. **E** Quantitative analysis of Runx2 and osteocalcin

To determine whether Fam53B gene expression was increased, quantitative real-time polymerase chain reaction (qRT-PCR) analysis and Western blot (WB) analysis were used for verification. After treating MC3T3-E1 mouse precursor osteoblasts with 1 µg/mL cerium oxide nanoparticles for 48 h, we observed a significant increase in Fam53B expression (Fig. 4a). Subsequently, we determined whether Fam53B bridged CeO_2_ NPs and β-catenin signaling. We treated MC3T3-E1 cells with different combinations of β-catenin inhibitor (KYA1797K) and CeO_2_ NPs for 48 h. As expected, Fam53B significantly increased the nuclear accumulation of β-catenin and the expression of downstream osteogenic genes OCN and Runx2. KYA1797K partially reversed the effect of CeO_2_ NPs on osteogenic differentiation (Fig. 4b–d).

## Discussion

The role of CeO_2_ NPs in osteogenic differentiation has been partially demonstrated in previous studies. Lu et al. disclosed that CeO_2_ NPs promote osteogenic differentiation of bone marrow mesenchymal stem cells by activating ERK pathway [14]. Vascular invasion is the premise of bone remodeling and fracture repair. Xiang et al. stated that CeO_2_ NPs promote proliferation, differentiation, and angiogenesis of endothelial progenitor cells by increasing the stability of HIF-1αin mesenchymal stem cells and increasing vascular endothelial growth factor expression [15]. However, the mechanism of CeO_2_ NPs in precursor osteoblasts has not been elucidated.

Although no human pharmacokinetic studies have been performed, CCK-8 analysis suggested that 0–3 µg/mL CeO_2_ NPs had no significant effect on cell viability of MC3T3-E1 precursor osteoblasts. The influence of 1 and 3 µg/mL CeO_2_ NPs on promoting osteogenesis and its mechanism were further investigated in subsequent experiments. Alizarin Red S staining was performed first. Extracellular matrix mineralization is a major part of bone formation; consequently, the number and size of calcium nodules can be used to measure the degree of osteoblast differentiation. The results demonstrated that CeO_2_ NPs significantly enhanced the extracellular matrix mineralization of MC3T3-E1 precursor osteoblasts, especially at 1 µg/mL. Runx2 and osteocalcin are significant marker genes for osteogenic differentiation, and their expression is increased at different osteogenic differentiation stages [16, 17]. Col Iα1 is a major component of bone matrix and is secreted by osteoblasts. Our study demonstrates that CeO_2_ NPs significantly upregulated their expression.

**Fig. 5:**
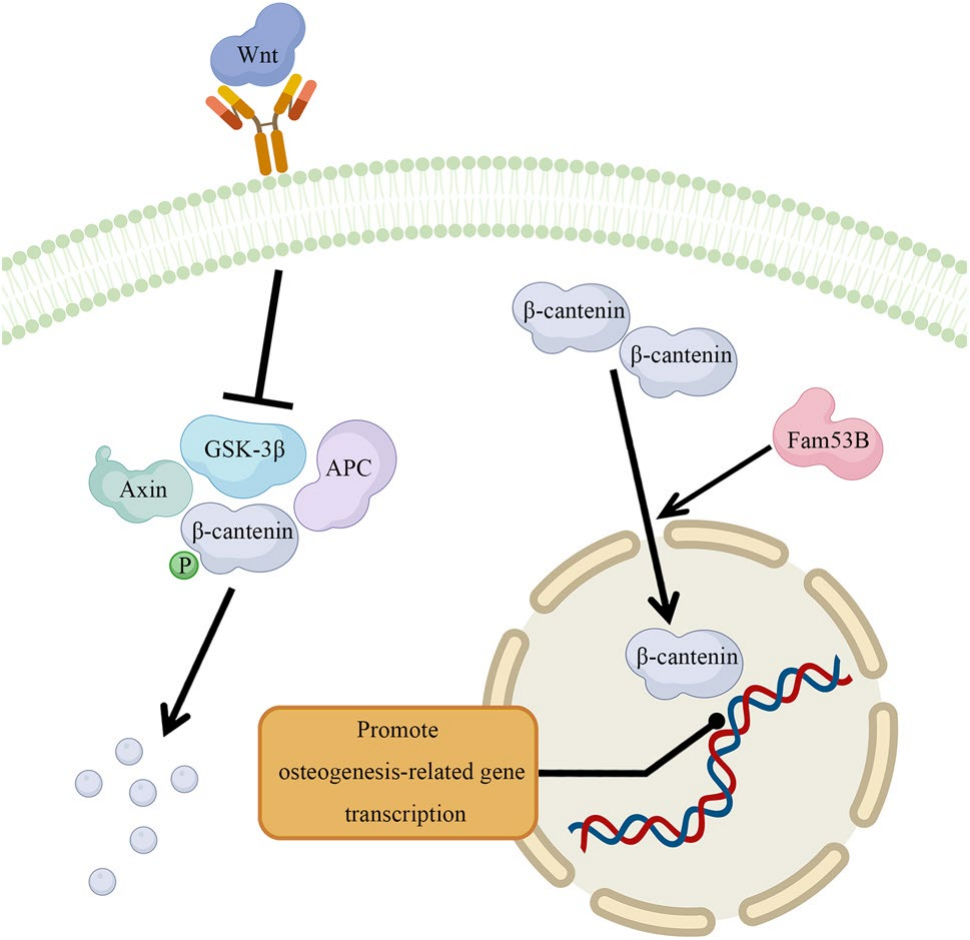
Potential mechanism of cerium oxide nanoparticles promoting osteogenesis. β-catenin proteins mediate the canonical Wnt pathway. In the cytoplasm, β-catenin is phosphorylated by the complexes of GSK-3β, APC, and Axin and is subsequently degraded. Wnt stimulation inhibited GSK-3β activity, resulting in β-catenin accumulation in the cytoplasm, which translocates into the nucleus later and promotes the transcription of target genes. Cerium oxide nanoparticles promoted β-catenin nuclear translocation by promoting Fam53B protein expression

Based on previously mentioned results, we suggested that 1 µg/mL CeO_2_ NPs had the best effect. Therefore, it was used to treat MC3T3-E1 precursor osteoblasts, and total RNA was extracted for mRNA-seq. The results demonstrated that Fam53B gene expression in MC3T3-E1 precursor osteoblasts significantly increased after CeO_2_ NPs. After treating MC3T3-E1 mouse precursor osteoblasts with CeO_2_ NPs, we detected a significant increase in Fam53B expression. Thermes et al. found that the absence of Fam53B reduced cell proliferation in medaka fish [18]. Kizil et al. found that Fam53B loss in zebrafish cells reduced nuclear accumulation of β-catenin, a major protein downstream of Wnt pathway, while Fam53B overexpression promoted the nuclear translocation of β-catenin [13]. The bone morphogenetic protein, Wnt, Indian hedgehog, and other proteins are involved in differentiating precursor osteoblasts [19, 20]. The Wnt/β-catenin signaling pathway regulates osteogenic differentiation, bone formation, and the occurrence of various bone metabolic diseases [21]. The canonical Wnt pathway is initiated by binding Frizzled transmembrane receptors, low-density lipoprotein receptor-related protein 5 (LRP5), and/or LRP6 bind to secreted Wnts, resulting in activated dishevelled proteins [22]. Subsequently, activated dishevelled phosphorylates glycogen synthase kinase-3β (GSK-3β), inhibiting its function. β-catenin protein was released from the complex of GSK-3β, adenomatous polyposis coli (APC), and axin. Then, β-catenin protein accumulates in the cytoplasm and translocates into the nucleus. Using T cell factor/lymphoid enhancing factor (TCF/LEF), β-catenin can activate the expression of c-myc, c-Jun, and other downstream transcription factors, thus promoting the expression of osteogenic genes [23]. In addition, β-catenin increased Runx2 expression (Fig. 5). KYA1797K can promote β-catenin degradation. After adding KYA1797K to the culture medium, we found that promoting MC3T3-E1 precursor osteoblasts differentiation by CeO_2_ NPs was abolished.

This study also has certain limitations. We only evaluated the effect of CeO_2_ NPs in vitro, lacking data in vivo experiments. Therefore, in future studies, we will further evaluate the role of CeO_2_ NPs in vivo.

## Conclusion

To summarize, CeO_2_ NPs promoted Fam53B expression, which increased the nuclear accumulation of β-catenin, thus activating Wnt pathway. Wnt pathway activation contributes to increased expression of downstream osteoblast-related genes, promoting the differentiation of MC3T3-E1 precursor osteoblasts.

## Data Availability

Not applicable.
